# Identification of new inhibitors against human Great wall kinase using *in silico* approaches

**DOI:** 10.1038/s41598-018-23246-0

**Published:** 2018-03-20

**Authors:** Ummi Ammarah, Amit Kumar, Rajesh Pal, Naresh C. Bal, Gauri Misra

**Affiliations:** 1 Amity Institute of Biotechnology, Amity University, Noida, 201313 U.P. India; 2 Department of Mechanical, Chemical and Materials Engineering, University of Cagliari, via Marengo 2, 09123 Cagliari, Italy; 3 Modeling and Simulations group, Center for advanced study research and development in Sardinia (CRS4), Loc. Piscina Manna, 09010 Pula, Italy; 4 KIIT University, Bhubaneshwar, Orissa India

## Abstract

Microtubule associated serine/threonine kinase (MASTL) is an important Ser/Thr kinase belonging to the family of AGC kinases. It is the human orthologue of Greatwall kinase (Gwl) that plays a significant role in mitotic progression and cell cycle regulation. Upregulation of MASTL in various cancers and its association with poor patient survival establishes it as an important drug target in cancer therapy. Nevertheless, the target remains unexplored with the paucity of studies focused on identification of inhibitors against MASTL, which emphasizes the relevance of our present study. We explored various drug databases and performed virtual screening of compounds from both natural and synthetic sources. A list of promising compounds displaying high binding characteristics towards MASTL protein is reported. Among the natural compounds, we found a 6-hydroxynaphthalene derivative ZINC85597499 to display best binding energy value of −9.32 kcal/mol. While among synthetic compounds, a thieno-pyrimidinone based tricyclic derivative ZINC53845290 compound exhibited best binding affinity of value −7.85 kcal/mol. MASTL interactions with these two compounds were further explored using molecular dynamics simulations. Altogether, this study identifies potential inhibitors of human Gwl kinase from both natural and synthetic origin and calls for studying these compounds as potential drugs for cancer therapy.

## Introduction

Cell cycle regulation requires an intricate balance of various kinases and phosphatases. Studies have attempted to understand the regulation of kinases during cell division still leaving several gaps^1^. Important proteins involved in mitotic regulation include Cyclin B-Cdk1 and Greatwall kinase (Gwl). The human orthologue of Gwl kinase is known as Microtubule associated serine/threonine-like kinase (MASTL), which is encoded by MASTL gene and regulates the mitotic entry in mammalian cells^2–4^. Identification of Gwl in *Drosophila* followed by studies in *Xenopus* egg extracts established that the activation of Gwl kinase results in the inhibition of an important phosphatase, namely PP2A-B55, responsible for the dephosphorylation of mitotic substrates leading to their exit from the mitotic cycle^2,5,6^. Same research groups further identified the substrate of Gwl as c-AMP regulated phosphoprotein 19 (Arpp19). Phosphorylation of Arpp19 by Gwl is important for PP2A-B55 inhibition and thus in the entry to mitotic phase. Recent studies have shown that phosphatases such as PP1, PP2A and PP1R3B dephosphorylate MASTL, thus regulating the cell cycle in humans promoting mitotic exit^7,8^. Another protein regulated by Gwl is α-endosulfine (ENSA) but the exact role of this protein in the cell cycle is highly debated^1^. Arpp19 and ENSA are proposed to inhibit B55δ subunit of PP2A during mitotic (M) phase which is essential to keep cyclin B1-CdK1 activity high^9,10^. The Gwl/ ENSA pathway links metabolic responses to cell cycle control, as demonstrated by budding and fission yeast studies^1,11^. Studies in *Xenopus* egg extracts have established its role in DNA damage recovery in late G2 phase. It further regulates the activation of CDK1 after the removal of damaged DNA^12^. Depletion of MASTL is reported to cause severe mitotic phenotypes, such as aneuploidy, defects in chromosome condensation, and failure to inactivate the spindle assembly checkpoint, with consequent defects in chromosome segregation and cytokinesis^13,14^.

Structurally, MASTL is classified as a member of the AGC family of kinases which consists of about 60 kinases including PKA, PKG, PKC, etc. that play an important role in the regulation of cell division, growth, metabolism, and differentiation^15,16^. It is a unique AGC kinase, which unlike most AGC kinases is devoid of a hydrophobic motif despite the presence of a hydrophobic pocket that specifies its distinctive mechanism of regulation^6^. It has a distinct T-loop region with the insertion of about 500 amino acids. However, MASTL is much less explored in comparison to other AGC kinases. It has been established that MASTL is phosphorylated during mitosis and this phosphorylation is critical for its activation. MASTL activity is believed to be stimulated by binding of its hydrophobic pocket with the hydrophobic motif of other AGC kinases, such as Rsk2, provided the linker residue (Ser-875) of MASTL is phosphorylated^6,17^.

Upregulation of MASTL is associated with various types of cancers including breast, prostate and oral cancers that correlates with the recurrence of tumor in patients suffering from head and neck squamous cell carcinoma. Further, it suppresses the functioning of DNA damage responsive genes thus increasing the susceptibility to DNA damage induced cell proliferation. Previous studies have shown that knocking down MASTL in breast cancer, head and neck squamous cell carcinoma cell lines make them more susceptible to chemotherapy treatments circumventing the resistance problems. Cancer treatment involves both chemotherapy and radiation resulting in DNA damage^12^. Normal cells have cellular DNA damage repair systems that identify and repair the damaged DNA. It has been reported that cancerous cells with increased levels of MASTL developed resistance to the treatment, thereby enhancing the possibility of tumor recurrence^18^. Thus, all these studies have established MASTL as an important therapeutic target in cancer^19^. Elucidation of molecular mechanisms underlying cancer progression is important for cancer therapeutics. Chemotherapy has dominated cancer therapeutics for a long time, but recently kinase inhibitors have also been proven efficient and quite reliable for cancer treatment. More than 25 kinase inhibitors have been approved for cancer therapy, and numerous others are under clinical trials^19,20^. However, in comparison to other kinases such as PLK1 and Aurora kinases, MASTL is less studied. Inhibition of MASTL targets certain cancer types causing cell death, without harming normal cells^18,21^.

*In silico* analysis for studying protein-ligand interactions has successfully been applied in biochemical research^22,23^, which has revolutionized the techniques of drug designing, moving the pharmaceutical industry to the forefront of targeted drug discovery. With the advent of virtual screening approach, huge libraries of chemical compounds can be explored rapidly to identify the structures of potential small molecules that bind to specific sites of target molecules, usually a protein receptor or an enzyme. In view of the significant role of MASTL in promoting correct timing of mitosis and its emergence as a novel drug target, our aim is to identify potential natural and synthetic compounds with significant inhibitory potential against the MASTL protein. To date, there is a single compound named GKI-1 that is proposed as a first line inhibitor against this protein^24^. New assays are developed to screen kinase specific libraries for the search of potential Greatwall inhibitors^25^. A dearth of knowledge in the area of MASTL inhibition has been the driving force behind the present study. The compounds that can specifically inhibit this kinase will be of immense importance in cancer therapy.

**Figure 1:**
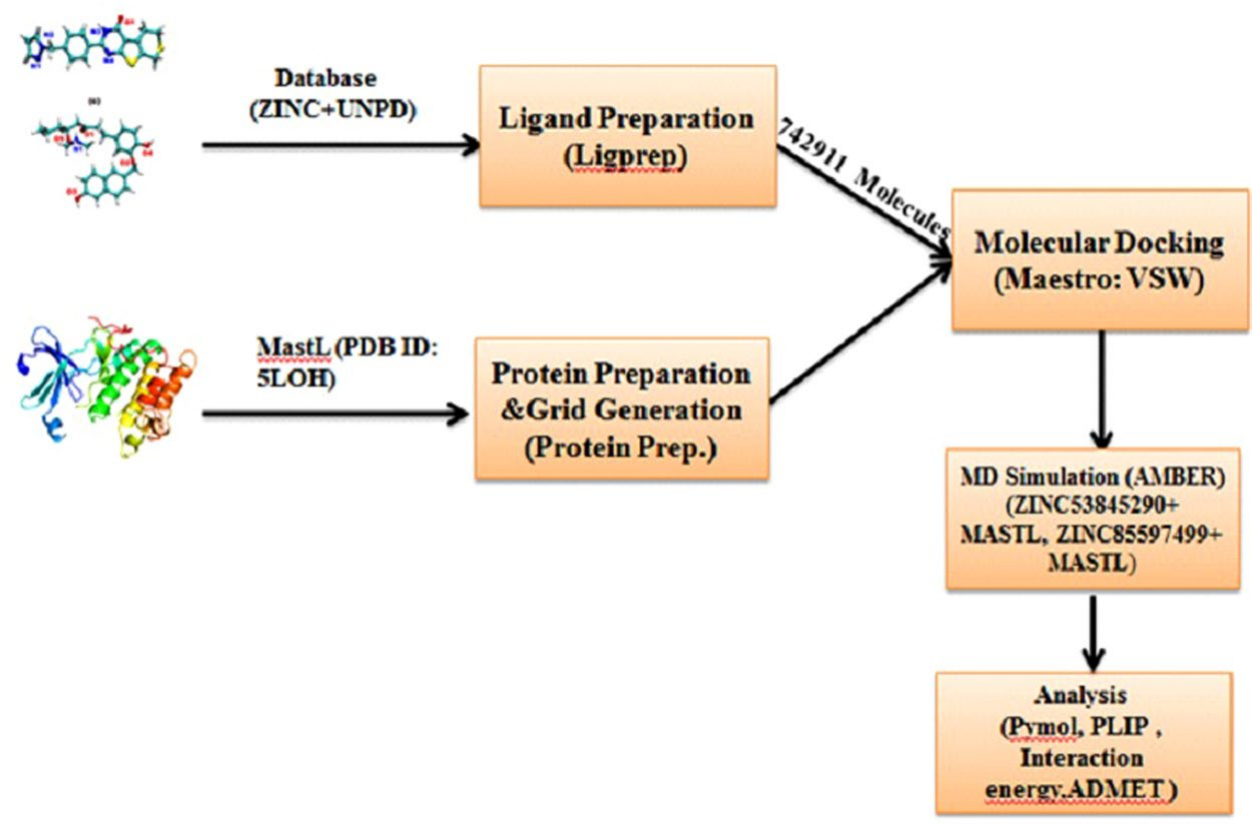
Graphical representation of the overall workflow.

In the present work, virtual screening of several compounds derived from both synthetic and natural sources were executed with the objective of identifying potential inhibitors against MASTL protein. Docking studies provided ten hits that exhibited good binding potential with good MMGBSA score. The interaction of the two best compounds namely (5R)-1-[4-hydroxy-3-[(6-hydroxy-2-naphthyl) methoxy] phenyl]-5-(methylaminomethoxy) octan-3-one (natural) and 2-[4-(1H-Pyrazol-1-ylmethyl)phenyl]-3,5,6,8-tetrahydro-4H-thiopyrano[4′,3′:4,5]thieno[2,3-d]pyrimidin-4-one (synthetic) were studied using all atom molecular dynamics simulations. The natural compound is a 6-hydroxy naphthalene derivative, while the synthetic compound is a thieno-pyrimidinone based tricyclic derivative. Noncovalent forces play an important role in stabilizing the protein-ligand interactions^26^. We performed a systematic analysis of protein-ligand interactions for the best compounds and physicochemical properties that influence the ligand binding characteristics were investigated. A flowchart summarizing the present study is shown in Fig. 1. The results presented in this study hold immense importance as the compounds identified can be studied further for their *in vitro* and *in vivo* efficacy. We anticipate this study to be providing a starting point for initiating design of more effective MASTL inhibitors.

## Results

### Docking studies

Recently solved crystal structure of the kinase domain of Gwl (PDB ID: 5LOH) has been used for docking studies. The protein structure was further modeled to include the missing disordered C-helix (activation loop) of the kinase from the X-ray. Thus, docking studies were performed with both the reported crystal structure and also our modeled structure to evaluate the role of activation loop in binding. To date, the only inhibitor identified against MASTL protein is GKI-1, which binds in the active site of the protein^24^. Nevertheless, the mechanism of action of this compound, its potency and efficacy against Gwl needs further study^24^. Different databases namely UNPD^27^, TCM Database Taiwan^28^, AfroDb and ZINC databases^29^ were explored for the virtual screening of potential compounds. About 536,525 synthetic compounds and 206,413 natural compounds from databases were subjected to virtual screening approach, which resulted in the identification of about 5 potential hits from the synthetic repository (Table 1, Supplementary Table S1) and five compounds from the natural sources (Table 2, Supplementary Table S2) exhibiting inhibitory potential. A list of 100 synthetic (Supplementary Table S3) and 51 natural compounds (Supplementary Table S4) have also been provided in the supplementary file for further experimental investigation (Supplementary Information).

**Table 1:** List of synthetic compounds showing better binding energies against MastL.

ZINC ID	DOCK SCORE (−kcal/mol)	MMGBSA	MOL. WEIGHT	H. BOND
ZINC53845290	−8.369	−86.562	380.4	2
ZINC77292085	−10.232	−84.409	486.58	7
ZINC20201746	−9.355	−81.369	363.476	2
ZINC01029685	−8.441	−78.002	396.509	1
ZINC77891226	−8.646	−77.742	356.811	4

**Table 2:** List of natural compounds showing better binding energies against MASTL.

**ZINC ID**	**DOCKING SCORE**	**MMG-BSA**	**MOL. WEIGHT**	**H. BOND**
ZINC85597499	−10.093	−81.975	451.5	4
UNPD178438	−9.233	−80.133	482.4	6
ZINC14679203	−9.268	−78.996	342.4	4
UNPD218939	−9.098	−78.973	412.4	6
UNPD72628	−9.334	−77.605	394.3	3

MMGBSA binding energies are approximate free energies of binding and a more negative value indicates stronger binding. MMGBSA binding energies (reported in kcal/mol) for the compounds were calculated to rank them; and only those compounds that displayed a better binding score against MASTL protein with respect to compound GKI-1^24^ are reported in Tables 1 and 2. We further found that binding of the compound to the modeled MASTL structure containing the activation loop did not display any significant changes in binding energy values with respect to the X-ray structure (Supplementary Table S5). Furthermore, the compounds exhibit no new interaction with this loop, including GKI-1, which was considered as a reference for our study. We describe below the nature of interaction network between the five synthetic and five natural compounds with the MASTL protein, obtained from docking.

**Figure 2:**
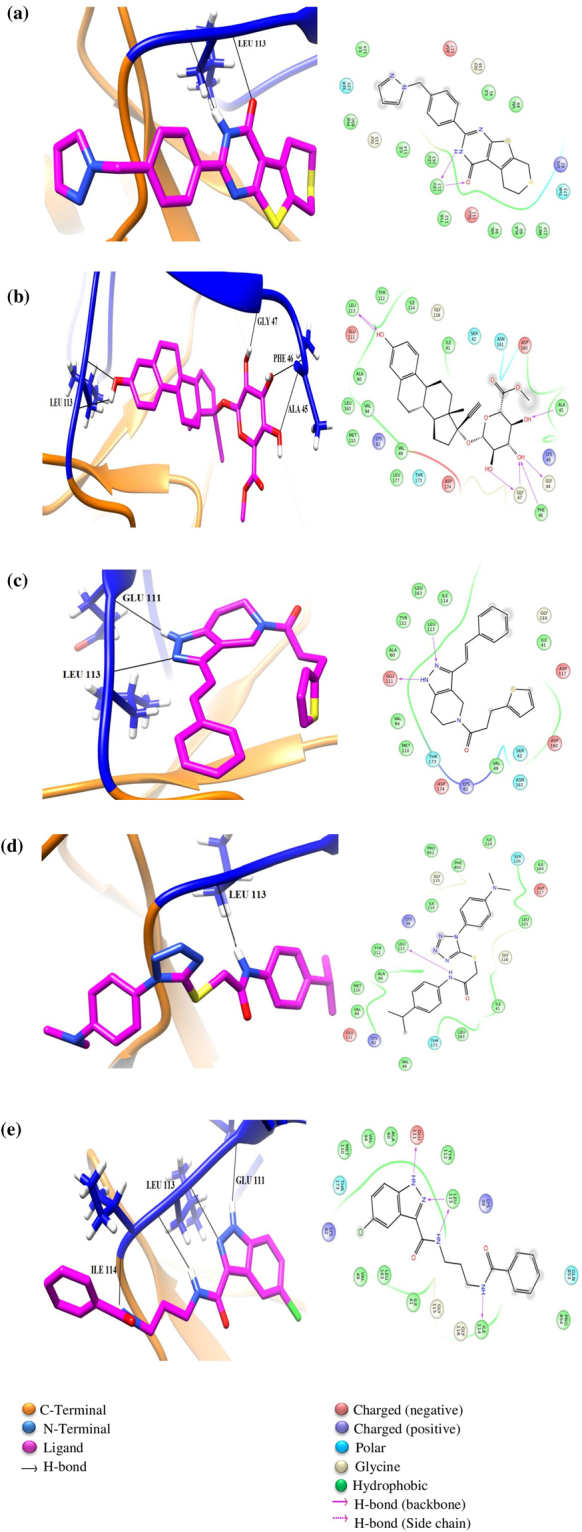
Docked poses of top 5 synthetic compounds. The N and C terminal lobe are represented in blue and orange color respectively. (**a**) ZINC53845290 (**b**) ZINC77292085 (**c**) ZINC20201746 (**d**) ZINC01029685 (**e**) ZINC77891226.

Among the synthetic compounds, compound with ZINC ID: ZINC53845290, displayed the best MMGBSA energy. ZINC53845290 is a dithiazine three membered cyclic structure and forms interactions with oxygen atom of the hydroxyl group and adjacent alpha nitrogen of the pyrimidine ring (Fig. 2a). In detail, the oxygen atom of the hydroxyl group at position 4 of the pyrimidine ring forms hydrogen bond with backbone nitrogen of residue Leu113, while the nitrogen at position 3 of the pyrimidine ring forms hydrogen bond with backbone oxygen atom of residue Leu113 (Fig. 2a). The next compound ZINC77292085, which is structurally characterized with four hydroxyl groups, is a Ethynyl Estradiol 17-|A-D-Glucuronide Methyl Ester (which is a sterol attached to a sugar moiety), displayed the second best MMGBSA energy. The hydroxyl group present in ring A of the estradiol skeleton was found to be involved in hydrogen bond interactions with backbone oxygen atom as well as amine group of residue Leu113 (Fig. 2b), while the hydroxyl groups at position 2 and 3 of the glucuronide formed hydrogen bonds to backbone oxygen of residue Gly 47. In particular, we note the hydroxyl group at 3^rd^ position to participate in hydrogen bond interactions with backbone oxygen in residue Gly44 and hydrogen atom of backbone amine group in residue Phe46. Further, the oxygen atom of the hydroxyl group at position 4 is exhibiting hydrogen bond interaction with the hydrogen atom of the backbone amine group in residue Ala45 (Fig. 2b). The third compound ZINC20201746 is phenylethenyl-1,4,6,7-tetrahydropyrazolo[4,3-c] pyridin-5-yl]-3-thiophen-2-ylpropan-1-one. In this case, we found the two conjugated nitrogen atoms at position 1 and 2 of the central pyrazole ring to display hydrogen bond interactions with oxygen atom of backbone in residue Glu111 and hydrogen atom of amine group in residue Leu113, respectively (Fig. 2c). The fourth compound ZINC01029685 is 2-[1-[4-(dimethylamino)phenyl]tetrazol-5-yl]sulfanyl-N-(4-propan-2-ylphenyl)acetamide, a unique compound composed of two benzyl moieties held together by a sulfide bridge. In this case, we observed nitrogen atom of the acetamide moiety to be involved in hydrogen bond with backbone oxygen atom of residue Leu113 (Fig. 2d). The fifth synthetic compound ZINC77891226 is N-(3-benzamidopropyl)-5-chloro-1H-indazole-3-carboxamide. In this case, the nitrogen 2 of the pyrazole ring and the nitrogen of the adjacent amide displayed hydrogen bond interactions with backbone amine group present in residue Leu113, respectively. On the other hand, the hydrogen in the imine group of pyrazole ring formed hydrogen bond with the backbone oxygen atom of residue Glu111, and the benzamide nitrogen at the other end interacted with the backbone oxygen of residue Ile114 (Fig. 2e). In summary, among all the synthetic compounds we note backbone nitrogen and oxygen of Leu113 to be commonly involved in hydrogen bonding interactions with the compounds, thus suggesting it as a hot spot residue involved in interaction with the inhibitors. Other than the hydrogen bonds, we also noted residues Leu163, Val94 and Ala60 involved in hydrophobic interactions in all the synthetic compounds.

**Figure 3:**
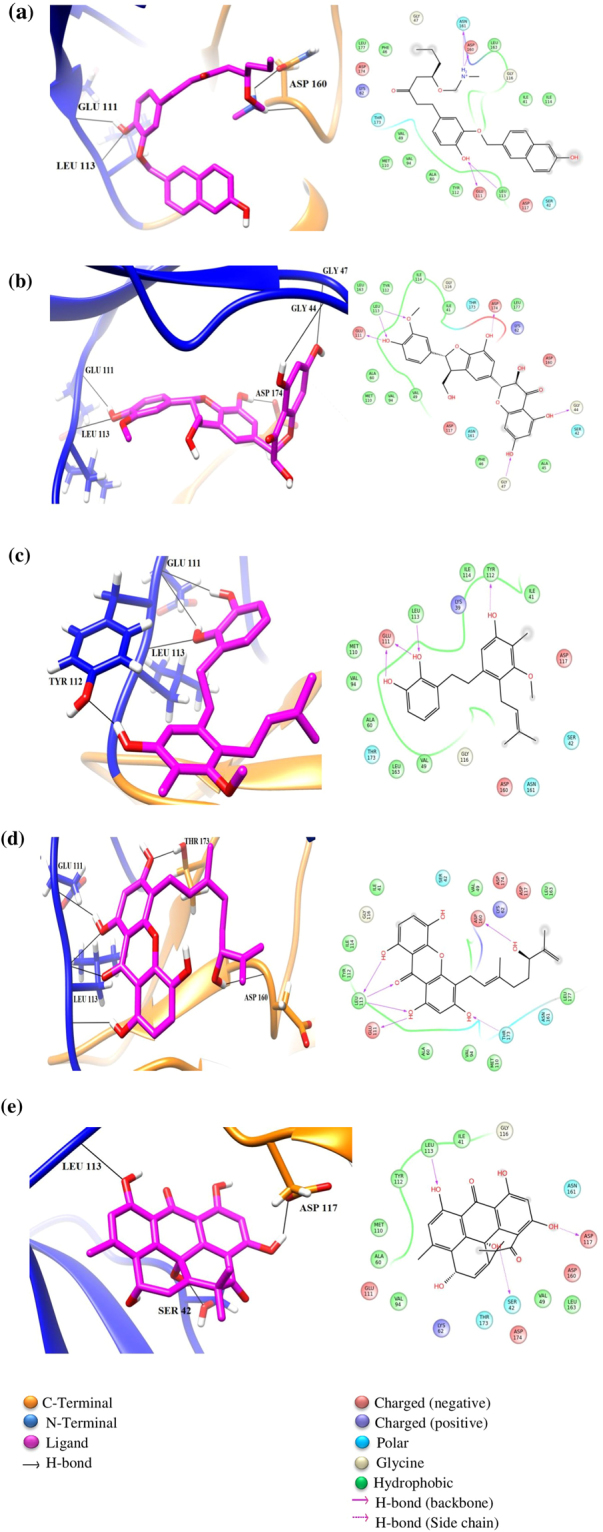
Docked poses of top 5 natural compounds. The N and C terminal lobe are represented in blue and orange color respectively. (**a**) ZINC85597499 (**b**) UNPD178438 (**c**) ZINC14679203 (**d**) UNPD218939 (**e**) UNPD72628.

The natural compounds exhibiting good binding characteristics were obtained after virtual screening using TCM^28^ and UNPD^27^ databases. Among the natural compounds, compound ZINC85597499, with chemical name (5R)-1-[4-hydroxy-3-[(6-hydroxy-2-naphthyl)methoxy]phenyl]-5-(methylaminomethoxy)octan-3-one, displayed the best MMGBSA binding energy. The four hydroxyl groups of the tri- substituted central phenyl ring of the compound ZINC85597499 formed hydrogen bonds with oxygen atom of backbone in residue Glu111 and amine of residue Leu113. While, the nitrogen of the methyl amino methoxy group displayed hydrogen bond interaction with the oxygen atoms of side chain carbonyl in residue Asn161 and backbone oxygen of Asp 160 respectively (Fig. 3a).

The second compound UNPD178438 has a chemical name (+)-silychristin|(2R,3R)-3,5,7-trihydroxy-2-[(2R,3S)-7-hydroxy-2-(4-hydroxy-3-methoxyphenyl)-3-hydroxymethyl-2,3-dihydrobenzofuran-5-yl]chroman-4-one|SC|Silychristin|silychristin A|silychristine, with three key structural groups: benzopyran, benzofuran and phenyl. A rich network of interactions was observed between its three key structural groups and MASTL residues. In detail, the oxygen atom of the hydroxyl group attached to the C7 position of the benzopyran moiety was hydrogen bonded to the backbone amine group in residue Gly47, while the hydroxyl group at C5 position formed hydrogen bond with oxygen atom of backbone in residue Gly 44. The hydroxyl group attached to the C7 position of the central benzofuran structure displayed hydrogen bond interaction with oxygen atom of side chain carboxyl group in residue Asp174. Finally, the oxygen atoms of C4 hydroxyl group and C3 methoxy group of the terminal phenyl moiety were found to exhibit hydrogen bond interactions with hydrogen of backbone amine group in residue Leu113 and also interacted with oxygen atom of backbone in residue Glu111 (Fig. 3b). The third natural compound ZINC14679203 is 3-[2-[5-hydroxy-3-methoxy-4-methyl-2-(3-methylbut-2-enyl)phenyl]ethyl]benzene-1,2-diol. We note the hydroxyl group attached at 1,2- benzene-diol position to interact with oxygen atom of backbone in residue Glu111 while the 2-hydroxyl group interacts with hydrogen of backbone amine moiety in residue Leu113, and the hydrogen of 5-hydroxy group of the central penta-substituted benzene ring interacts with oxygen atom of phenol ring present at the side chain of residue Tyr112 (Fig. 3c). The fourth natural compound UNPD218939 is 4-(3,7-dimethyl-6-hydroxy-2,7-octadienyl)-1,3,5,8-tetrahydroxyxanthone| garcihombronone B, and interacts with carboxyl group and hydrogen at the backbone of Leu113 residue through hydroxy-1, hydroxy-8 and carbonyl-9 of the xanthene moiety. Besides, hydroxy-1 of the same compound interacts with oxygen atom of backbone in Glu111. While, oxygen atom in hydroxy-3 of xanthene interacts with the side chain hydroxy group in residue Thr173 and hydroxy-6 of the octane-diene tail interacts with the backbone oxygen atom of residue Asp160 (Fig. 3d). The fifth natural compound UNPD72628 with chemical name Resistoflavin and characterized by a para hydroxyl group in the central ring was found to interact with the side chain of residue Ser42. While, the hydroxyl group at the C1 position of Naphthanthrone ring A and C6 position of ring C interacted with the backbone of residue Leu113 and the side chain oxygen atom of carboxyl group in residue Asp117, respectively (Fig. 3e).

Interestingly, all the five natural compounds bind to the same active site as reported for the GKI-1, thus indicating similar mechanism of action against MASTL protein.

### ADMET properties

Several important pharmacokinetic parameters need to be essentially considered before taking the hits for further experimental screening. A lucid understanding of these properties prevents late stage failure of compounds in clinical trials^30^. The permissible QPlogPo/w values, which reflect better absorption and permeability characteristics of the compounds, were evaluated (Tables 3 and 4). Interestingly, our top-ranked synthetic compound (ZINC53845290) and natural (ZINC85597499) compound displayed better absorption and permeability characteristics, between the other selected compounds. Moreover, the calculated QPlogS values indicated better absorptivity, cell permeability and oral absorption profile for the top-ranked compounds derived from synthetic (Table 3) and natural sources (Table 4), respectively.

**Table 3:** ADMET study of synthetic chemical compounds.

Compounds	QPlog Po/w	QPlogS	QPPCaco	% oral absorption
ZINC53845290	4.3	−6.684	1343.53	100
ZINC77292085	2.9	−5.781	102.77	80.02
ZINC20201746	4.3	−5.642	715.84	100
ZINC01029685	4.2	−6.874	473.53	100
ZINC77891226	3.6	−5.604	398.60	94.67
Recommended Values	−2 to 6.5	−6.5 to 0.5	<25 poor >500 Great	<25% poor >80% high

**Table 4:** ADMET study of natural compounds.

**Compounds**	**QPlog Po/w**	**QPlogS**	**QPPCaco**	**% oral absorption**
ZINC85597499	4.3	−5.586	99.344	88.444
UNPD178438	1.2	−4.896	10.893	40.095
ZINC14679203	4.2	−4.606	1134.142	100
UNPD218939	3.1	−4.256	76.729	79.128
UNPD72628	1.8	−3.913	36.846	66.086
Recommended Values	−2 to 6.5	−6.5 to 0.5	<25 poor >500 Great	<25% poor >80% high

QPlogPo/w: Predicted octanol/water partition co-efficient log P

QPlogS: Predicted aqueous solubility; S in mol/L

QPPCaco: Predicted Caco-2 cell permeability in nm/s.

### MD simulation analysis

**Figure 4:**
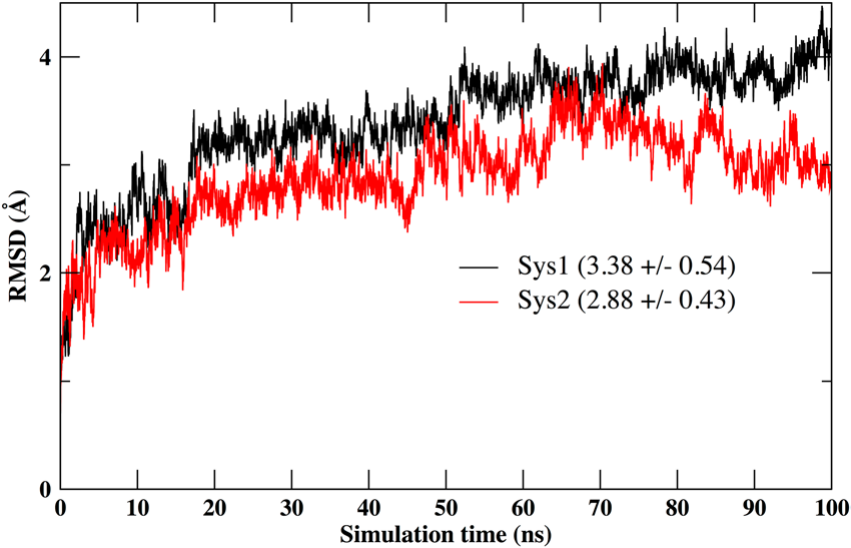
RMSD plot of C-alpha atoms of protein residues for system 1 (Sys1) and system 2 (Sys2).

The top ranked synthetic compound ZINC53845290 and the top ranked natural compound ZINC85597499 complexes were subjected to 100 ns of MD simulations. We refer to the synthetic compound ZINC53845290 as ligand 1 (Lig1), natural source compound ZINC85597499 as ligand 2 (Lig2). System 1 is the MASTL protein in complex with the synthetic compound ZINC53845290 and the system 2 is MASTL in complex with the natural compound ZINC85597499. The stability of protein-ligand complexes was evaluated by calculating the root mean square deviation (RMSD) values for the C-alpha atoms of residues during MD simulation (Fig. 4). We observed the RMSD deviation value to be slightly higher for the system 1 in comparison to system 2. Thus, indicating a much stable interaction with the natural compound. The convergence of MD simulations was estimated using Good Turning statistical approach^31^ (Supplementary Information).

### Hydrogen bond interactions

**Figure 5:**
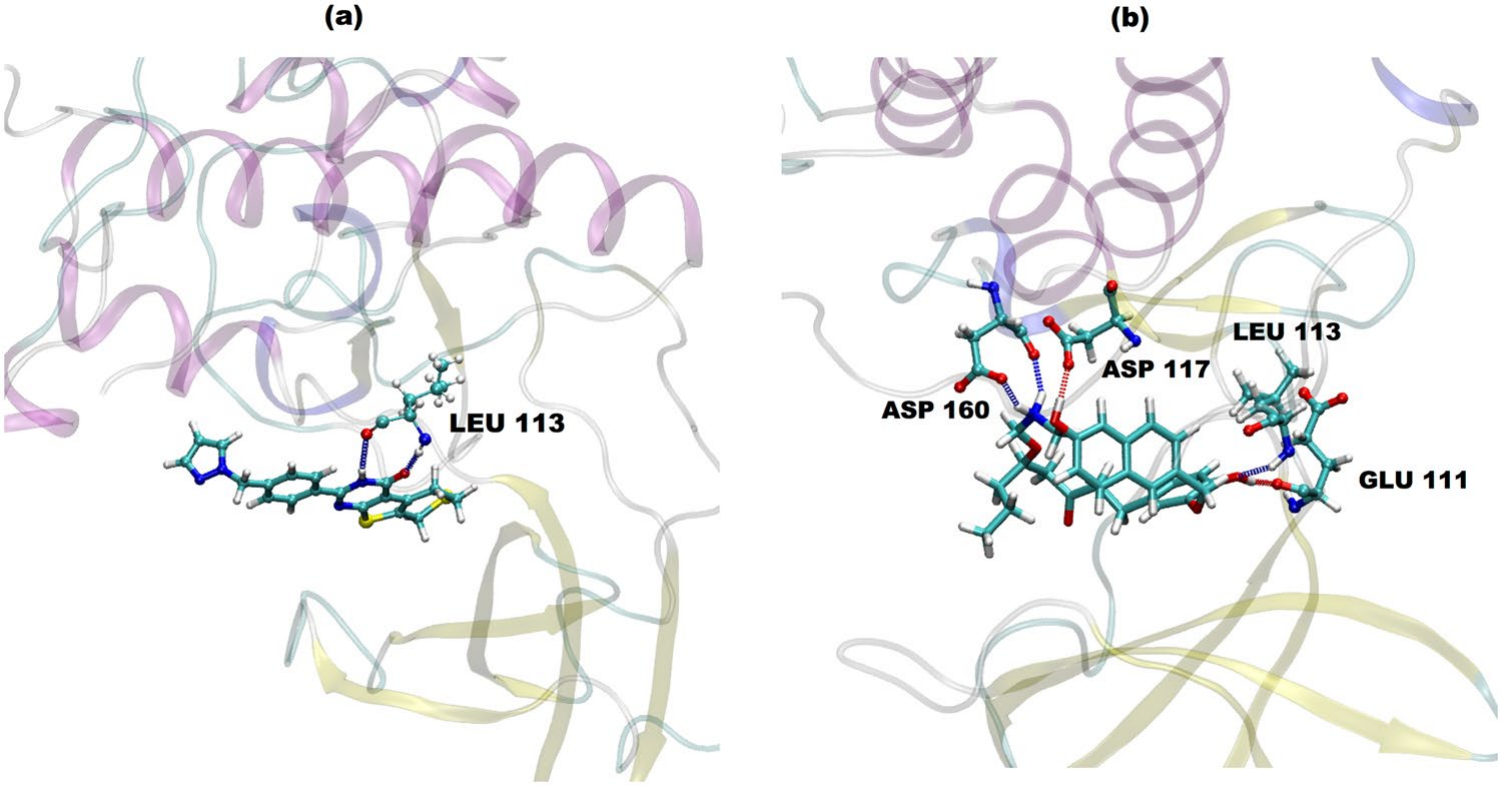
H-bond interactions. (**a**) Ligand 1 complex and in (**b**) ligand 2 complex. The protein residues involved are shown in ball and stick representation.

We further analyzed hydrogen bonded (H-bond) interactions between protein and the ligands (Fig. 5). We report only persistent H-bond interactions, which are present for at least 20% of total simulation time. We found H-bond interaction between lig1 (ZINC53845290) and MASTL residue Leu113 (Fig. 5a), while four H-bonds for lig2 (ZINC85597499) complex system (Fig. 5b).

### Interaction energy calculations

**Figure 6:**
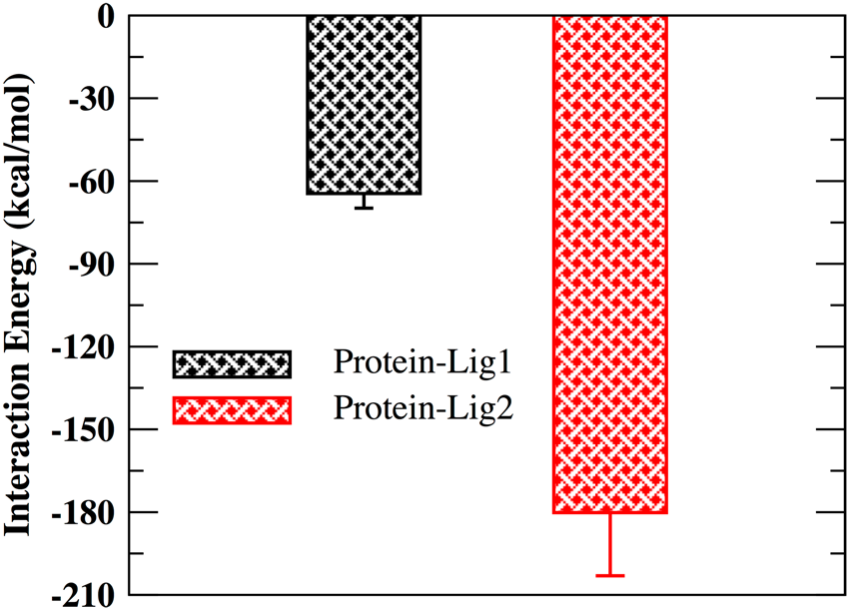
Interaction energy plot corresponds to non-bonded energy values comprising of Van der Waals and electrostatic energy between the protein and ligand.

The interaction energy term corresponds to the non-bonded energy values comprising of Van der Waals and electrostatic energy between the ligand and the protein residues (Fig. 6). A more negative value of interaction energy corresponds to better ligand binding characteristics. A more negative value of interaction energy was found for the lig2 complex system with respect to lig1 complex. Thus, indicating much favorable binding affinity of the protein complex towards lig2 (natural compound).

### Binding energy estimation using Solvated Interaction Energy (SIE)

**Figure 7:**
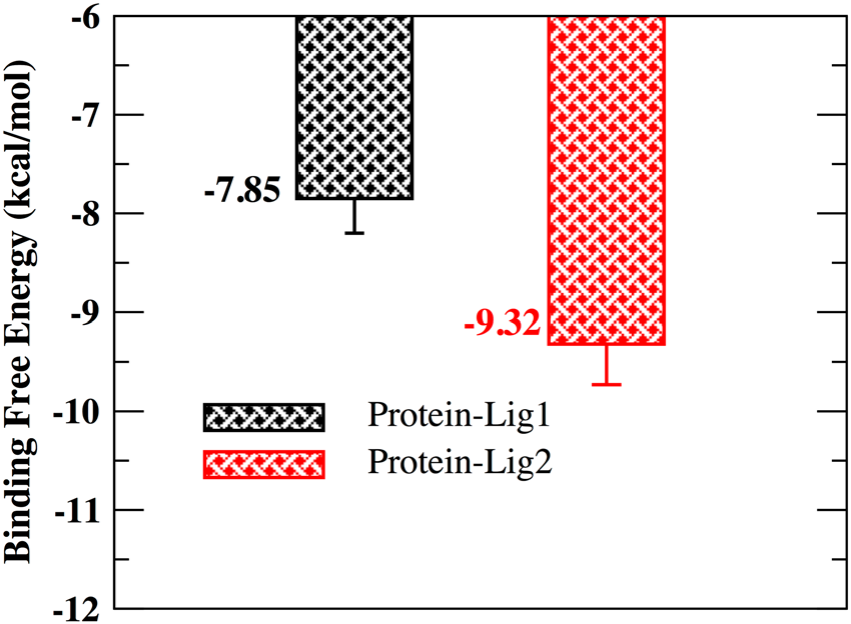
Binding free energy calculation using SIE approach.

The binding free energy for ligand-protein complex was calculated using solvated interaction energy (SIE) method^32^ incorporated within SIETRAJ software package^33^. SIE treats the protein-ligand system in atomistic detail and solvation effects implicitly similar to methodology used in MM-PBSA approach. However, it also incorporates a crude but effective treatment of entropy–enthalpy compensation in the binding energy estimation. The SIE binding free energy value was calculated at a time interval of 20 ps from 100 ns simulation trajectory. In Fig. 7, we report the average binding free energy value for the two ligand-protein complexes. We observe lig2 to display much favorable binding free energy (−9.32 kcal/mol) towards protein (MASTL) with respect to ligand 1 (−7.85 kcal/mol).

## Discussion

One of the approaches exploited for cancer prevention is the prevention of cell cycle progression in tumour cells. Uncontrolled cell division is the hallmark of cancer progression. Thus, several cell cycle regulating proteins have proved to be an effective therapeutic target for cancer prevention. Inhibitors against Aurora A, Aurora B and Plk1^34^ kinases have been continuously evaluated for their potential to function as anticancer agents^35^. Another group of important kinases that have been extensively explored on the similar lines are cyclin-dependent kinases (cdks) and other cyclins. It is encouraging to note that several compounds such as PD0332991, Flavopiridol, SNS-032, Indisulam, SNS-032, Seliciclib, Bryostatin-1, AZD5438, and SCH 727965 are in various stages of clinical trials, which successfully inhibit these proteins^36^. Moreover, these compounds are also effective in combating drug resistance. However, combinatorial therapy has been proved to be more successful as compared to the single compound administration specifically in solid tumours^36^.

The quest for new therapeutic targets in cancer therapy has driven the attention towards MASTL kinase protein. Indeed, MASTL is upregulated in different types of cancers by playing a crucial role in mitotic progression. Owing to its role in progression of cancer and recurrence of tumour^18^, MASTL has been established recently as a new therapeutic target. In a recent study^24^, the authors have successfully determined the structure of the GWL minimal kinase domain and designed a small-molecule inhibitor GKI-1, which displayed cellular efficacy in the treatment of cells by reducing the substrate ENSA/ARPP19 phosphorylation levels. Moreover, the same authors pointed GKI-1 to be promising towards development of more potent and selective GWL inhibitors. The GKI-1 being the only inhibitor reported against MASTL protein calls for further improvisation to enhance its potential and exploration to decipher its mechanism of action^24^. In this scenario, identification of potential inhibitors against a key mitosis regulating protein becomes crucial.

In this study, in our first step, virtual screening of compounds derived from both synthetic and natural sources included in various databases was performed. We identified five potential compounds each from synthetic and natural sources and subsequently performed molecular docking studies on the compounds with the X-ray structure of MASTL protein. Molecular docking techniques are a powerful computational tool to predict identification of target sites of the ligand and the protein molecule^37–39^. Our docking studies results indicated the identified compounds (listed in Tables 1, 2, S1 and S2) to show a better MMGBSA score with respect to the reported compound GKI-1. However, the docking approach does not allow complete incorporation of protein’s flexibility. Therefore, to improve the accuracy of the predicted docked pose and to include the protein flexibility, we performed all atom MD simulations for the top ranked synthetic (ZINC53845290, lig1) and natural (ZINC85597499, lig2) protein-compound complexes. In detail, the synthetic compound (lig1) is a tricyclic with a thieno-pyrimidinone scaffold and natural compound (lig2) is 6-hydroxynaphthalene derivatives. The therapeutic implication of naphthalene derivatives as anti-inflammatory compounds^40^ and antioxidative agents for the prevention of tumor progression is well established^41^. On similar lines, tricyclic derivatives are reported in multitude of conditions ranging from allergy, viral infections, central nervous system pathologies, inflammation, cancer to cardiovascular diseases^42,43^. Their role in the prevention of multi drug resistant cancer has recently been studied^44^. The stability of the protein-compound complexes were found to be stabilized by non-covalent interactions such as hydrogen bonding, Van der Waal and electrostatic. An abundant interaction picture in lig2 complex with respect to lig1 complex resulted in a better interaction energy value (Fig. 6), better binding free energy value (Fig. 7) for lig2 (ZINC85597499) with respect to lig1 (ZINC53845290).

To address the role of disordered C-helix or activation loop of the kinase, we remodelled the protein structure to include the missing disordered C-helix (activation loop) of the kinase and repeated docking and MD simulations. However, no significant change in the energetics of protein-compound binding was observed with respect to MD simulations without the C-helix (Supplementary Information).

Further, to allow a correct and fair comparison between standard GKI-1 compound reported in literature and the identified compounds in the present study, we performed docking and MD simulations for GKI-1 complex (Supplementary Information). Docking studies indeed revealed same ligand binding site for the reference GKI-1 compound and the two lead compounds (lig1, lig2). Backbone nitrogen and oxygen of Leu113 was found to participate in persistent hydrogen interactions in all the three cases (Fig. 5, Supplementary Information). The calculated interaction energy value between modelled MASTL protein and the compound was found to be highest for lig2 (−165 kcal/mol), followed by an intermediate value of lig1 (−70 kcal/mol) and lowest for the reference compound GKI-1 (−51 kcal/mol). Moreover, the ADMET calculations for our lead compounds (lig1, lig2) reinforced our proposition that all these compounds have permissible logP values, aqueous solubility, membrane permeability and good oral absorption profile. In fact, the lig2 (natural compound) and lig1 (synthetic) that we have characterized in detail show the best oral absorption. Thus, ADMET properties clearly indicate towards the drug likeliness of these compounds.

In conclusion, computational outcomes presented in this study provide a promising foundation for further experimental inquiry and validation of the identified compounds as potential MASTL inhibitors.

## Materials and Methods

### Protein Preparation

The crystal structure of kinase domain of MASTL (PDB ID: 5LOH)^24^ was retrieved from Protein Data Bank (http://www.rcsb.org/pdb/home/home.do)^45^. Staurosporine was removed from the active site for docking various compounds of interest. The optimization and minimization of protein was then carried out using Protein Preparation Wizard tool in Schrodinger^46^ and OPLS-2005 force field^47^. The tool fixes the protein and makes it suitable for molecular docking. It corrects the incorrect bond orders, charge states, orientations of different amide, hydroxyl and aromatic groups within a protein structure, which cannot be determined by the X-ray structure due to limited resolution. To minimize the strains and steric collisions in protein, energy minimization was done by molecular mechanics calculation using OPLS-2005 force field^47^ available in the Protein Preparation tool^46^.

The missing disordered C-helix (activation loop) of the kinase from the X-ray structure was modeled using Schrodinger as well as online I- TASSER webserver^48^.

### Ligand Preparation

Docking was carried out using synthetic and natural compounds. Synthetic compounds were obtained from ‘drugs now’ subset of ZINC database^29^ and natural compounds were downloaded from UNPD database^27^ and different catalogues available in ZINC database^29^. About 536,525 synthetic compounds and 206,413 natural compounds were prepared using LigPrep tool^49^ which generates accurate and energy minimized 3-dimensional structures and also applies sophisticated rules in order to correct the Lewis structure and eliminates mistakes in the ligand structures.

### Active site and Grid generation

A grid was defined around the active residues using “Receptor Grid Generation” in Glide module of Schrodinger suite^50–52^. The grid enclosing the active site where Staurosporine inhibitor was bound was generated with an internal size of 14 × 14 × 14 (x × y × z, Å), and was large enough to accommodate the active site of the protein, in order to allow each ligand to search for the potential binding site. Docking was also carried out in predicted inhibitor site as predicted by the sitemap module in Schrodinger. The molecules docked in reported inhibitor site had better MMGBSA and Glide score as the final results were compared. Thus, the ligands were docked around the nucleotide-binding pocket of Great wall kinase. The amino acids present in the active site were Glu111 and Leu113 including Ile41, Val49, Ala60 and Leu163 from the N-lobe, and Thr173 and Leu163 from the C-lobe.

### Molecular Docking/Virtual Screening

Protein-ligand docking studies were carried out based on the optimized structures of kinase domain of MASTL. Both the crystallographic and modeled structures were used for the docking purpose. A total of 742,938 molecules have been docked (characterization of molecules). Virtual Screening Workflow performs docking of a large collection of compounds against one or more targets. It uses Glide docking at 3 accuracy levels, HTV, SP, and XP^50–52^.

### ADMET analysis

It predicts the adsorption, distribution, metabolism, excretion, and toxicity of any compound.

We have used Qik Prop module^53^ inbuilt in Schrodinger for studying these properties of the best compounds (both synthetic and natural as summarized in Tables 1, 2, S1 and S2).

### MD simulations

**Figure 8:**
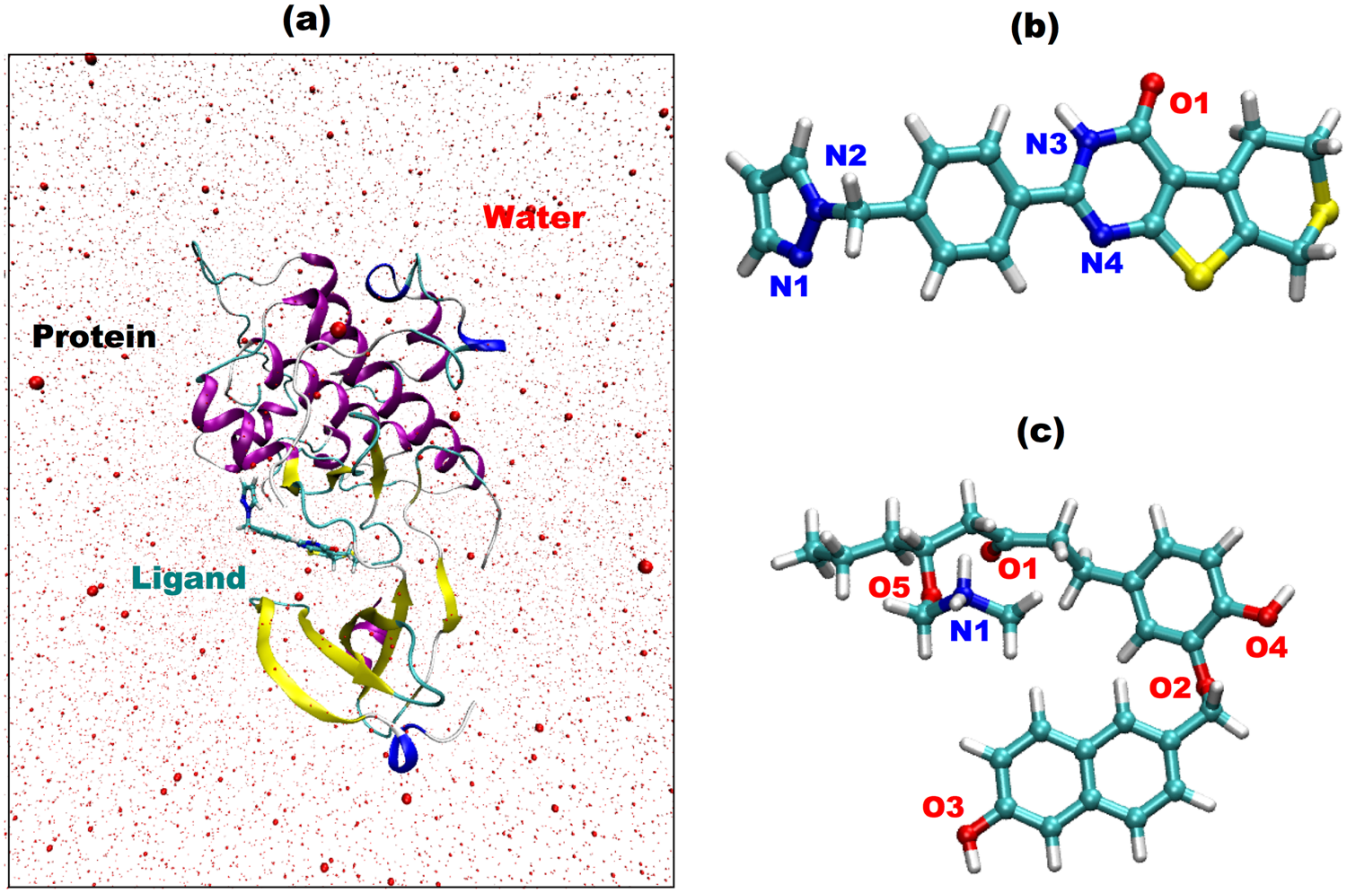
Solvated Protein-Ligand complex. The two ligands investigated in this work are shown in (**b**) and (**c**). Compound (**b**) is ZINC53845290 (**c**) is ZINC85597499.

The charges and the force-field parameters for the ligand were obtained following the standard AMBER protocol^54^. The protein-ligand structures obtained from docking experiments were chosen as starting structures for molecular dynamics simulations. We used tleap module of Amber 11 software package to build solvated and neutral protein-ligand molecular systems. The protein-ligand complex was inserted in a water box with a minimum distance between any atom of the complex and edge of the box of 20 Å (Fig. 8). Subsequently, counter-ions were added to neutralize the two systems. TIP3P parameters for water molecules and for Amber 99 force-field parameters for protein residues were used. The initial dimensions of the simulation box edges were [92 102 98], and the total numbers of molecules in the protein-ligand molecular system were – System1 83120 atoms and System 2 83152 atoms. All the simulations were performed using AMBER 99 force field parameters and using NAMD software^55^.

The Molecular systems were energy minimized and gradually heated to 300 Kelvin (K) in steps of 30 K with positional constraints of 50 kcal mol^−1^ Å^−2^ on carbon alpha atoms for a simulation time of 0.2 ns. The positional constraints on the carbon alpha were then slowly released in steps of 10 kcal mol^−1^ Å^−2 ^^56^. After an initial relaxation and equilibration run for 3 ns, a long production run for a simulation length of 100 ns for the two molecular systems was performed. For the long-range electrostatic interactions we used particle mesh Ewald scheme and with a cut-off radius 12 Å was employed for the non-bonded interactions^57^.

The hydrogen bonded (H-bond) interactions between protein residue pairs were calculated using a geometrical criterion, with a donor-acceptor cut-off distance of 3.1 Å and donor-hydrogen-acceptor cut-off angle 130 degree^56^. The interaction energy between the protein residues and ligand atoms was calculated by evaluating the non-bonded energy values comprising of Van der Waals and electrostatic energy, using the energy plugin of Namd software^57,58^. To provide an estimate for the convergence of our simulations, along with standard RMSD calculations we also adapted a novel Good -Turing statistical approach proposed by Koukos and Glykos^31^. This method allowed us to estimate the probability distribution, p_unobserved_ (RMSD), of unobserved configurations as a function of RMSD distance between unobserved and observed molecular configurations in MD simulations (Supplementary Information).

### Binding Free Energy Calculations

The binding energy for the protein-ligand complexes was evaluated using the solvated interaction energy (SIE) method. In the SIE method, the binding energy (DeltaG_bind_) in aqueous solution is approximated by (i) an interaction energy contribution (E_inter_), and (ii) a desolvation free energy contribution (DeltaG_desolv_), which resemble the formalism used in MM-PBSA^59^. Even though entropy is not included explicitly, calibration of the obtained SIE free energy is done using an empirically determined parameter, obtained by fitting a training set of 99 protein–ligand complexes, thus allowing a crude but effective treatment of entropy–enthalpy compensation. Embedding empirical information from structural databases, SIE proves to be quite a robust method for providing quantitative affinities. SIE method was used to rank binding strengths, and the binding energy values obtained by SIE method was used to classify the binding characteristics of the two ligands. This technique also has been employed recently to evaluate binding free energy in different biological systems^57,60,61^.

## Electronic supplementary material

Supplementary information
