# Supplementary material for: Identification of new inhibitors against human Great wall kinase using in silico approaches
Source: Sci Rep. 2018 Mar 20;8:4894. doi: 10.1038/s41598-018-23246-0 (PMC5861128; doi:10.1038/s41598-018-23246-0)
Supplement: Supplementary file 1 — Supplementary information [file 41598_2018_23246_MOESM1_ESM.pdf]

Supplementary Information

Identification of new inhibitors against human great wall kinase using *in silico* approaches

Ummi Ammarah[1*], Amit Kumar,[2,3*] Rajesh Pal[1], Naresh C. Bal[4], Gauri Misra[#1]

[1]Amity Institute of Biotechnology, Amity University, Noida-201313 (U.P.), India

[2] Department of Mechanical, Chemical and Materials Engineering, University of Cagliari, via Marengo 2, 09123 Cagliari, Italy

[3] Modeling and Simulations group,, Center for advanced study research and development in Sardinia (CRS4), Loc. Piscina Manna, 09010 Pula, Italy

[4] KIIT University, Bhubaneshwar, Orissa, India

*These authors should be considered joint first authors

# Corresponding author

Dr. Gauri Misra

Assistant Professor

Amity Institute of Biotechnology

Amity University

Noida- 201313 (U.P.), India

Email: kamgauri@gmail.com, gmisra@amity.edu

Table S1. Chemical structures of top synthetic compounds

ZINC ID	TWO DIMENSIONAL STRUCTURE
ZINC53845290	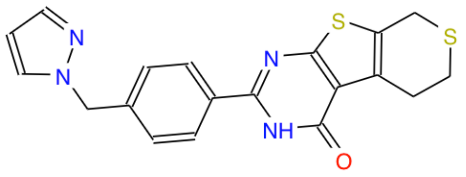
ZINC77292085	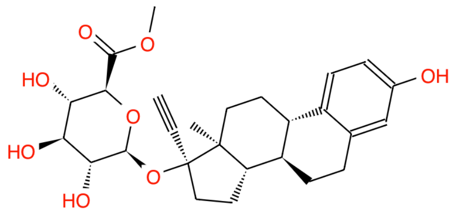
ZINC20201746	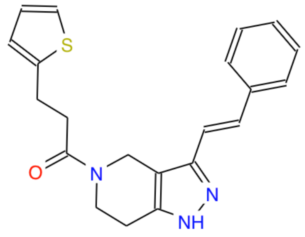
ZINC01029685	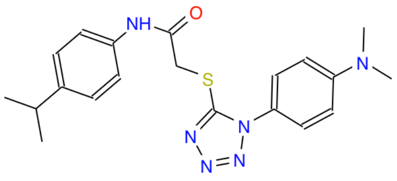
ZINC77891226	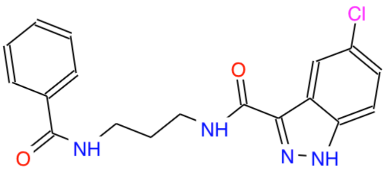

Table S2. Chemical structures of top Natural compounds

**Chemical ID**	**TWO DIMENSIONAL STRUCTURE**
**ZINC85597499**	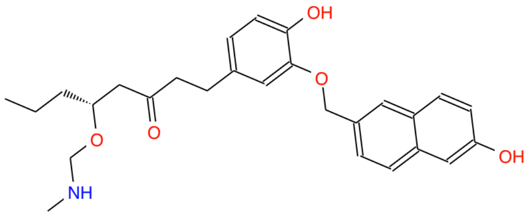
**UNPD178438**	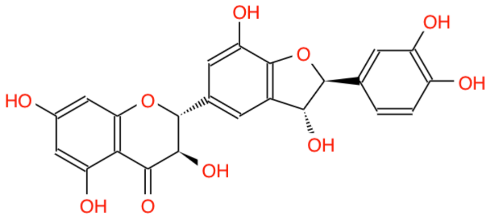
**ZINC14679203**	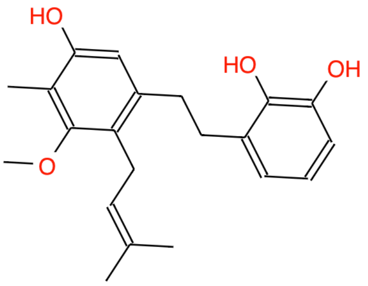
**UNPD218939**	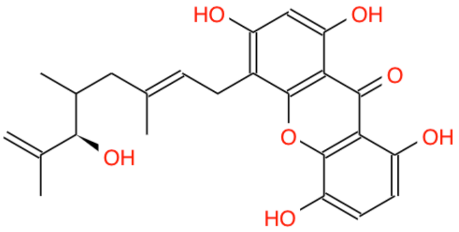
**UNPD72628**	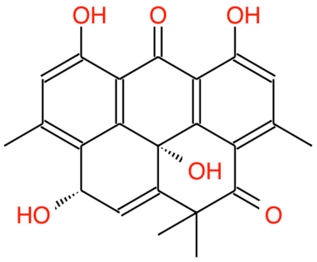

Table S3. List of top 100 synthetic chemical compounds sorted according to MMGBSA score.

S. No	ZINC ID`	Molecular Weight	Docking Score	MMGBSA Score
**1.**	ZINC53845290	380.482	-8.405	-86.562
**2.**	ZINC77292085	486.561	-10.232	-84.409
**3.**	ZINC77292081	472.534	-10.645	-82.552
**4.**	ZINC20201746	363.476	-9.355	-81.369
**5.**	ZINC01029685	396.509	-8.441	-78.002
**6.**	ZINC77891226	356.811	-8.646	-77.742
**7.**	ZINC82039371	360.379	-9.576	-77.691
**8.**	ZINC95474627	382.415	-9.976	-76.15
**9.**	ZINC15734996	368.511	-9.14	-76.115
**10.**	ZINC72430977	375.488	-9.372	-75.867
**11.**	ZINC09114348	371.41	-8.604	-75.551
**12.**	ZINC71747293	359.514	-9.481	-75.197
**13.**	ZINC72367862	331.435	-9.798	-74.409
**14.**	ZINC49906155	299.799	-8.848	-74.294
**15.**	ZINC12385459	412.503	-8.624	-74.181
**16.**	ZINC77891206	368.822	-11.086	-73.693
**17.**	ZINC71775810	356.467	-8.538	-73.031
**18.**	ZINC67895639	368.478	-8.448	-72.745
**19.**	ZINC49906156	299.799	-9.529	-72.686
**20.**	ZINC49921830	310.179	-9.425	-72.27
**21.**	ZINC82039371	360.379	-8.431	-72.133
**22.**	ZINC55183183	307.395	-10.081	-71.963
**23.**	ZINC95391460	367.837	-8.51	-71.715
**24.**	ZINC72172715	348.422	-8.78	-71.138
**25.**	ZINC37877074	312.195	-8.265	-70.718
**26.**	ZINC72168477	382.385	-8.893	-70.417
**27.**	ZINC77891209	404.855	-10.562	-70.408
**28.**	ZINC63856354	367.49	-8.335	-70.284
**29.**	ZINC82039448	367.41	-8.351	-70.247
**30.**	ZINC00902563	445.495	-8.334	-70.075
**31.**	ZINC71747294	359.514	-8.231	-69.537
**32.**	ZINC57836109	256.337	-8.175	-69.537
**33.**	ZINC72121111	363.418	-9.003	-69.508
**34.**	ZINC67472860	337.421	-8.307	-69.477
**35.**	ZINC41407476	272.346	-8.971	-69.467
**36.**	ZINC83379758	248.711	-8.76	-69.26
**37.**	ZINC77891233	305.781	-8.993	-69.049
**38.**	ZINC72367838	318.396	-8.569	-69.048
**39.**	ZINC78939374	333.448	-8.089	-68.664
**40.**	ZINC49906152	299.799	-8.633	-68.647
**41.**	ZINC95371588	264.37	-9.947	-68.414
**42.**	ZINC00351096	323.412	-8.338	-68.294

**43.**	ZINC71747294	359.514	-9.331	-68.156
**44.**	ZINC47423500	341.452	-8.378	-67.961
**45.**	ZINC16806569	311.337	-8.096	-67.891
**46.**	ZINC48356174	329.394	-10.428	-67.768
**47.**	ZINC83379749	248.711	-8.376	-67.653
**48.**	ZINC71877548	323.437	-9.06	-67.577
**49.**	ZINC49880190	283.326	-9.081	-67.534
**50.**	ZINC07738380	381.405	-8.6	-67.457
**51.**	ZINC19906806	266.299	-9.888	-67.218
**52.**	ZINC72135158	321.381	-10.436	-67.165
**53.**	ZINC71877549	323.437	-8.947	-67.023
**54.**	ZINC49906153	299.799	-9.126	-66.747
**55.**	ZINC49906157	299.799	-9.112	-66.726
**56.**	ZINC91646130	304.366	-9.591	-66.64
**57.**	ZINC71456788	262.351	-9.257	-66.543
**58.**	ZINC72151296	324.382	-9.727	-66.382
**59.**	ZINC41862983	327.385	-8.626	-66.348
**60.**	ZINC77891230	300.747	-9.207	-66.338
**61.**	ZINC55132374	330.429	-9.127	-66.29
**62.**	ZINC72151412	344.375	-8.285	-66.244
**63.**	ZINC97163749	334.215	-8.472	-66.232
**64.**	ZINC71973647	386.471	-8.34	-66.109
**65.**	ZINC39475640	298.744	-9.347	-65.999
**66.**	ZINC12880252	389.428	-8.88	-65.946
**67.**	ZINC77891234	308.767	-8.952	-65.863
**68.**	ZINC77322489	282.347	-9.253	-65.843
**69.**	ZINC77875845	377.445	-10.093	-65.791
**70.**	ZINC72367867	335.398	-9.055	-65.785
**71.**	ZINC23367688	376.457	-8.75	-65.738
**72.**	ZINC38716152	371.471	-8.537	-65.677
**73.**	ZINC71775747	300.316	-9.249	-65.584
**74.**	ZINC77891232	300.747	-9.041	-65.396
**75.**	ZINC95425678	298.772	-8.178	-65.374
**76.**	ZINC19906863	284.744	-8.51	-65.347
**77.**	ZINC77891234	308.767	-8.226	-65.291
**78.**	ZINC91690345	314.386	-9.515	-65.139
**79.**	ZINC00407344	292.355	-8.37	-65.012
**80.**	ZINC97111731	352.367	-8.636	-64.979
**81.**	ZINC72479074	315.392	-9.627	-64.85
**82.**	ZINC72361553	283.375	-9.845	-64.811
**83.**	ZINC06748472	306.32	-8.206	-64.805
**84.**	ZINC03896053	361.458	-8.396	-64.501
**85.**	ZINC95371589	264.37	-8.593	-64.458
**86.**	ZINC41407479	272.346	-8.401	-64.455
**87.**	ZINC97288761	294.358	-8.801	-64.448
**88.**	ZINC97813672	384.48	-8.645	-64.378
**89.**	ZINC41407482	272.346	-8.858	-64.201

**90.**	ZINC83543445	275.307	-8.846	-64.181
**91.**	ZINC49880191	283.326	-9.756	-64.105
**92.**	ZINC49891744	291.297	-8.82	-64.052
**93.**	ZINC77554626	378.433	-8.517	-63.958
**94.**	ZINC63845560	337.38	-8.313	-63.88
**95.**	ZINC49891793	291.321	-9.779	-63.69
**96.**	ZINC95425716	323.384	-8.848	-63.622
**97.**	ZINC38008420	301.79	-8.104	-63.578
**98.**	ZINC78657349	260.722	-8.116	-63.472
**99.**	ZINC55183184	307.395	-8.396	-63.404
**100.**	ZINC72172639	322.753	-8.265	-63.364

Table S4. List of top 51 natural compounds sorted according to MMGBSA score.

S. No	ZINC ID`	Molecular Weight	Docking Score	MMGBSA Score
**1.**	ZINC85597499	451.561	-10.093	-81.975
**2.**	UNPD178438	482.443	-9.754	-80.133
**3.**	ZINC14679203	342.434	-9.268	-78.996
**4.**	UNPD218939	412.438	-10.268	-78.973
**5.**	UNPD72628	394.38	-9.402	-77.605
**6.**	UNPD58243	346.336	-9.992	-76.172
**7.**	UNPD29541	354.359	-9.458	-75.455
**8.**	UNPD1518	455.469	-8.968	-74.316
**9.**	UNPD34722	380.232	-9.221	-72.041
**10.**	UNPD44499	354.359	-8.549	-71.623
**11.**	UNPD21346	334.282	-9.037	-71.555
**12.**	UNPD29649	360.406	-9.134	-70.655
**13.**	ZINC13340783	298.381	-9.339	-70.134
**14.**	ZINC14647251	332.266	-9.334	-69.777
**15.**	ZINC13383128	370.358	-9.367	-69.2
**16.**	UNPD39689	326.262	-9.734	-68.695
**17.**	ZINC13377892	376.449	-9.367	-68.492
**18.**	UNPD2163	334.325	-9.293	-68.191
**19.**	UNPD194804	346.379	-9.176	-68.154
**20.**	UNPD123349	394.216	-9.314	-68.061
**21.**	ZINC33833981	334.325	-9.527	-67.917
**22.**	UNPD160846	292.288	-9.555	-67.906
**23.**	UNPD156694	334.325	-9.368	-67.06
**24.**	UNPD71688	314.294	-9.248	-66.899
**25.**	ZINC14724692	334.325	-9.9	-66.839
**26.**	UNPD104607	339.347	-9.213	-66.195
**27.**	UNPD214879	316.267	-9.084	-65.588
**28.**	UNPD138280	290.272	-9.308	-65.176
**29.**	UNPD17298	290.272	-8.752	-64.973

**30.**	ZINC85626701	416.47	-10.735	-62.969
**31.**	UNPD182356	304.299	-8.442	-61.422
**32.**	UNPD53461	286.24	-9.904	-61.213
**33.**	UNPD170510	330.293	-9.091	-60.835
**34.**	ZINC14725222	286.24	-8.972	-60.587
**35.**	UNPD125720	302.326	-9.658	-60.326
**36.**	UNPD136612	228.247	-9.049	-59.926
**37.**	UNPD4180	334.282	-8.814	-59.85
**38.**	ZINC03861630	254.242	-8.864	-59.117
**39.**	UNPD222884	288.256	-8.82	-58.879
**40.**	UNPD99088	300.31	-9.046	-58.77
**41.**	ZINC14814114	284.268	-8.979	-58.666
**42.**	UNPD161790	240.215	-9.089	-57.675
**43.**	ZINC03860369	240.215	-9.089	-57.655
**44.**	ZINC13340729	274.273	-9.023	-56.554
**45.**	UNPD200162	362.379	-9.195	-55.76
**46.**	ZINC06523948	302.24	-8.911	-55.66
**47.**	UNPD139628	288.299	-9.133	-51.518
**48.**	UNPD223245	336.298	-9.364	-50.355
**49.**	UNPD84698	418.313	-10.004	-49.054
**50.**	UNPD439	260.246	-9.639	-44.219
**51.**	UNPD99785	323.345	-8.635	-42.695

Table S5. Interaction energy and binding free energy values (in kcal/mol) for the compounds bound to modeled and X-ray protein complexes.

Lead Compounds	Interaction Energy (kcal/mol)		Binding Free Energy (kcal/mol)	
	X-RAY	MODEL	X-RAY	MODEL
ZINC53845290 (Lig1)	-65 ± 6	-70 ± 8	-7.85 ± 0.41	-7.43 ± 0.47
ZINC85597499 (Lig2)	-180 ± 22	-165 ± 16	-9.32 ± 0.58	-9.12 ± 0.62

Figures

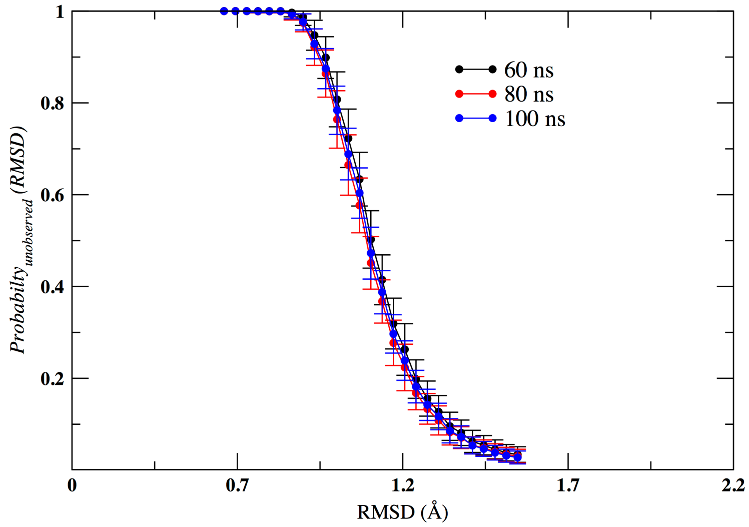

Figure S1. Good-Turing convergence test. Probability$_{unobserved}$ (RMSD) as a function of RMSD distance for ligand 2 complex system.

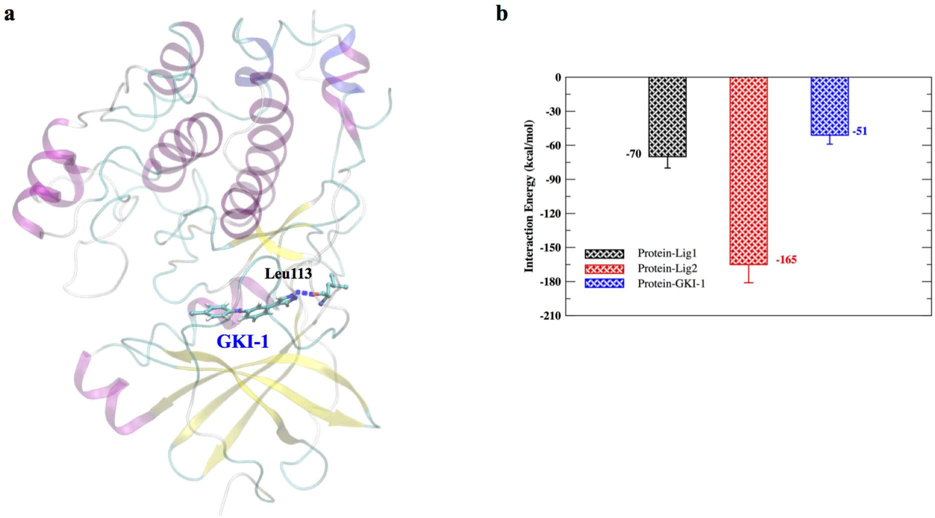

Figure S2. MASTL-GKI complex system. In (a) GKI-1 compound (in licorice) docked in MASTL protein (represented in cartoon) binding site. Persistent hydrogen bond interaction between residue Leu113 and GKI-1 is shown by dashed blue line. In (b) Comparative interaction energy plot for MASTL protein with modeled C-helix in complex with Lig1 (ZINC53845290), Lig2 (ZINC85597499) and GKI-1 (reference) compounds.
